# Legalised Causes of Crime
†On Certain Legalised Forms of Temptation as Causes of Crime. By John Roberton. Manchester: 1857.


**Published:** 1857-07

**Authors:** 


### LEGALISED CAUSES OF CRIME.†

Mr. Roberton's essay is closely argued. His remarks on legalised forms of temptation are limited to three:—1. The laws relative to bastardy and seduction, amongst women of the lower classes. 2. The laws authorising the Sunday traffic in the sale of intoxicating drinks. 3. Some things in the present state of the Poor Law tending to engender crime. Regarding the first of these clauses he says, "Not that the law should be made less stringent towards the woman, but that some punishment should be meted out to the man by whom seductions are brought about, the man being the aggressive animal in illicit intercourse." Regarding the second clause, he argues the propriety of the entire suppression of Sunday traffic in intoxicating liquors; and in respect to the third clause, he suggests that every guardian of the poor should at certain intervals—say twice in the year—visit the out-door poor in the district for which he has been elected, so as to obtain a correct esti-

† On Certain Legalised Forms of Temptation as Causes of Crime. By John Roberton. Manchester: 1857.

mate of their physical and moral condition. What are the Manchester men about, that John Roberton does not represent them in St. Stephen's? Such a man should surely have a voice beyond the sphere of one English city.

